# Neglected tropical diseases in conflict zones: devastating consequences of a lack of inclusion

**DOI:** 10.1093/inthealth/ihaf165

**Published:** 2026-05-08

**Authors:** Margriet den Boer, Koert Ritmeijer

**Affiliations:** Médecins Sans Frontières, Plantage Middenlaan, Amsterdam, 1018 DD Netherlands; Médecins Sans Frontières, Plantage Middenlaan, Amsterdam, 1018 DD Netherlands

In this editorial for International Health’s Special Issue on neglected tropical diseases (NTDs) in conflict settings, we reflect on a year of remarkable successes in NTD control amidst fierce global health challenges. As World NTD Day draws near, we find ourselves in a world heavily impacted by conflict and a sharp decline in financial support for the world’s poorest and most neglected populations. The landscape has changed for NTDs in the last years: while the World Health Organization’s (WHO’s) new roadmap for NTDs (2021–2030)^1^ was launched with optimism, backed by funding pledges and strong commitments, a setback in NTD control and elimination is now being experienced, with >100 million tablets for mass drug administration (MDA) for preventive chemotherapy (PC)-NTDs expiring in warehouses this year,^2^ along with a deterioration of health systems in many of the affected countries. These are threats that create opportunities for NTDs to spread and re-emerge. Yet the WHO reports that amidst a sharp decline in funding—a 41% decrease from 2018 to 2023^3^—as of 17 November 2025, 58 countries have eliminated at least one NTD, including 24 across the African continent.^4^

According to the Institute for Economics and Peace, the world is facing a violent conflict crisis. The total number of state-based conflicts is now higher than at any point since World War II and global peacefulness has declined again this year, with more countries experiencing internal and external conflict, higher militarization and large refugee flows than any time in the 17 previous years.^5^

South Sudan, the Democratic Republic of Congo (DRC) and Haiti feature among the 10 most fragile states in 2024,^6^ characterized by conflict, an inability to provide a minimum of public services, widespread population displacement and poverty. In this issue we read about MDA campaigns for PC-NTDs in the highly insecure and challenging contexts of these countries.

The article by Sommers et al.^7^ describes a pilot MDA with triple-drug therapy for lymphatic filariasis (LF) achieved a high coverage in a community in Haiti, a success largely attributed to strong community engagement. The article by Idraku et al.^8^ describes MDA campaigns for LF and onchocerciasis in Maban County in Upper Nile State in South Sudan, where >200 000 refugees live in the country’s largest refugee camps. Through coordination with all stakeholders and meticulous planning, near-complete coverage was reached, illustrating that there are real advantages to rolling out MDA in refugee camps.

When filariasis morbidity management and disability prevention was implemented in Maban County in 2025, high numbers of lymphoedema and hydrocele cases were found through active screening. Similarly, in the article by Mandro-Ndahura et al.^9^ we read about a survey in Kanyaruchinya Internally Displaced Persons Camp in North Kivu in the DRC that identified a striking number of NTD-related morbidities, including cases of lymphoedema. It was described that these people experienced extreme stigma and isolation, with in one case, a woman too ashamed to leave her shelter. The findings led to successful advocacy for national recognition of lymphoedema as a disability and its addition to the national community-based rehabilitation case definitions.

The focus of national programs for PC-NTDs is on treating and preventing the acute infection stage, while those suffering from NTD-related chronic disfigurement and disability still do not receive sufficient attention. A recent article by Mutapi et al.^10^ emphasizes that documenting the lived experiences of these people is essential to fully understand the extent of their challenges and develop strategies to support them. Mutapi et al.^10^ conclude that people with chronic manifestations of NTDs are among the most neglected of NTD sufferers. Many of the NTDs have disabling and disfiguring consequences: these include LF, onchocerciasis, trachoma, leishmaniasis, leprosy, Buruli ulcer and snakebite.^11^ Of the 178 countries considered endemic for at least one of the NTDs causing disability, as of 2023, only 33 countries have national guidelines for the management of these disabilities.^3^

All three aforementioned articles in this issue describe rolling out MDA programs in extremely challenging settings, and this is further illustrated by the article by Harding-Esch.^12^ Here, health workers and program managers from a number of conflict-affected African countries share first-hand accounts of the operational difficulties in highly insecure settings. A wide range of useful innovative coping strategies and key research areas were identified from these interviews.

The last article by Bestwick et al.^13^ is a systematic review of the literature on NTD programs in conflict. This review of 26 studies includes the so-called case-management NTDs, diseases requiring individualized diagnosis, treatment and care rather than MDA. The vector-borne visceral (VL) and cutaneous leishmaniases (CL) fall into this category. Nine of 26 reviewed studies were on CL. The incidences of VL and CL were shown to sharply increase during conflict.^14^ Intense conflict causes a doubling of risk of CL,^15^ the main reason being a breakdown of municipal services (waste collection) and the rubble of broken buildings that create a breeding ground for the sandfly vector as well as rodent parasite reservoirs. The CL burden is very high in countries like Afghanistan, Syria and Pakistan.^16^ These three countries were responsible for 72% of the global reported burden in 2023.^17^ CL cases are also high among refugees in neighbouring countries.^18^ Without treatment, CL can leave disfiguring or debilitating scars, causing tremendous psychological distress and social isolation.^19^ Access to treatment remains very poor in these countries. Médecins Sans Frontières (MSF) is offering CL treatment in Pakistan, with many thousands of patients presenting at its clinics each year.

VL incidence was shown to increase up to sixfold in countries with very high levels of conflict and terror.^13^ In East Africa, VL is prone to outbreaks, associated with massive population displacement and people seeking safety and foraging for food in the acacia forests where the sandfly vector is abundant. As VL results in high fatality rates if not treated within months, access to treatment is crucial. This is perhaps best illustrated by the first documented VL outbreak in Western Upper Nile, South Sudan, during a period of intense armed conflict and famine. Based on seven retrospective community-based mortality surveys, MSF estimated that approximately 100 000 people died of VL between 1984 and 1994, which was one-third of the total population.^20^

Unfortunately, the case-management NTDs that need resource-intensive efforts to detect and treat cases in remote areas have proven less appealing to donors than the PC-NTDs. Support has been patchy and far from adequate. East Africa bears by far the greatest burden of VL, but although a regional elimination strategy has been developed^21^ and a landmark memorandum of understanding between countries was signed during the 2025 World Health Assembly,^22^ support by external donors has not been forthcoming. Many of the highly endemic VL countries (Sudan, South Sudan, Ethiopia, Somalia) are afflicted by armed conflict, creating conditions for VL to spread, while access to treatment deteriorates. Support for VL control programs in East Africa is currently only offered by the END Fund, while the WHO provides medicines and other support^23^ and MSF offers VL diagnosis and treatment in its field hospitals in Sudan, South Sudan and Ethiopia. US funding cuts have disrupted human immunodeficiency virus (HIV) and tuberculosis (TB) services, and as programs close and testing and treatment initiation decline, the expected increase in HIV and TB incidence in the next 5–10 y will exacerbate the spread and impact of VL through increased transmission and more severe disease.

The NTD prevalence and burden are highest in sub-Saharan Africa, accounting for more than one-third of the global burden.^1^ African hotspots have seen the largest escalation of violence in the last few years. Increasingly, NTDs occur in a setting of large-scale displacement, a collapse of water, sanitation and hygiene and health systems, stockouts of essential medicines, widespread malnutrition and extreme poverty. The question arises whether NTD elimination is feasible in such settings. The answer is that it was never more important. The articles in this issue, showing the courage, inventiveness and resilience of field workers administering MDA’s, persisting even in highly insecure settings, offer hope. MSF’s CL treatment sites in Pakistan and VL and snakebite treatment sites in Ethiopia, Sudan and South Sudan, situated close to areas of attack, and the continuing determined efforts of the national VL program in Sudan, supported by the END Fund, to reach patients are further examples of what is possible.

Human suffering due to conflict has perhaps never been more pronounced and visible for all to witness, more thoroughly documented, and our realization of inequity never more profound. Everything possible should be done to not compound this suffering by allowing NTDs to spread and cause further devastation in already extremely vulnerable populations.

## Data Availability

The data are available in the article.
